# Prevalence and antimicrobial susceptibility profiles of non‐typhoidal *Salmonella* isolated from chickens in Rajshahi, Bangladesh

**DOI:** 10.1002/vms3.440

**Published:** 2021-02-01

**Authors:** Bindu R. Sarker, Sumon Ghosh, Sukanta Chowdhury, Avijit Dutta, Liton Chandra Deb, Bidhan Krishna Sarker, Tania Sultana, Khandoker Mohammad Mozaffor Hossain

**Affiliations:** ^1^ Department of Veterinary and Animal Sciences, Faculty of Agriculture University of Rajshahi Rajshahi Bangladesh; ^2^ Infectious Diseases Division International Centre for Diarrhoeal Disease Research, Bangladesh (icddr,b) Dhaka Bangladesh; ^3^ Chittagong Veterinary and Animal Sciences University Chittagong Bangladesh; ^4^ Department of Public Health North Dakota State University Fargo ND USA

**Keywords:** antimicrobial resistance, Bangladesh, chickens, prevalence, *Salmonella*

## Abstract

Salmonellosis in poultry is an important disease that seriously impedes the development of the poultry industry. The increased resistance to antimicrobials against *Salmonella* has been a major public health concern worldwide. We conducted a study from January to June 2016 in and around the Rajshahi district of Bangladesh on the commercial chicken to isolate, identify and characterize poultry‐specific *Salmonella*, to assess the potential risk factors and to determine the antimicrobial resistance pattern of the isolates. The overall prevalence of *Salmonella enterica* was 41% (49/120) [95% CI: 31.95%–50.17%] with 41.7% in broiler chicken (25/60) [95% CI: 29.06%–55.12%] and 40% in layer chicken (24/60, 40%) [95% CI: 27.56%–53.46%]. Samples collected from Rajshahi city (OR = 1.37, 95% CI: 0.50–3.73) and Puthia Upazila (OR = 1.51, 95% CI: 0.56–4.12) were more likely to be positive for *Salmonella* than Charghat Upazila. *Salmonella* detection was 1.3 times higher in chicken, providing loose feed than those provided ready feed. All the isolates fermented dextrose, maltose and mannitol with the production of acid and gas, but did not ferment sucrose and lactose. The isolates showed catalase, MR, citrate utilization test and TSI agar test positive, but indole and V‐P tests negative. *Salmonella* isolates were sensitive to ciprofloxacin (90%), gentamycin (80%), amoxicillin (75%), streptomycin (70%), ampicillin (45%) and sulfamethoxazole‐trimethoprim (45%), whereas highly resistant to penicillin (100%) and nalidixic acid (100%) followed by sulfamethoxazole‐trimethoprim (55%), ampicillin (40%) and amoxicillin (25%). *Salmonella enterica* is endemic in commercial chicken production in Bangladesh with high prevalence. A considerable proportion of *Salmonella* isolates was found to be resistant to the majority of the common antimicrobial drugs. A good biosecurity system could be effective for the reduction of S*almonell*a. It is necessary to obtain universal commitments to establish prudent antibiotic use policies.

## Graphical abstract

This study reported the prevalence and antimicrobial susceptibility profiles of *Salmonella* isolated from chickens in Rajshahi, Bangladesh. This study showed the endemicity of *Salmonella* in commercial chicken production in Bangladesh with their resistant to the majority of the common antimicrobial drugs. *Salmonella* detection was 1.3 times higher in chicken, providing loose feed than those provided ready feed.

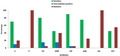

## 1 INTRODUCTION

Salmonella is an important food‐borne pathogen causing an estimated 153 million enteric infections and approximately 57,000 diarrhoeal deaths worldwide every year (Kirk et al., 2015).

Poultry and poultry product are often implicated as a potential risk factor for human salmonellosis (Bryan & Doyle, 1995; Humphrey, 2000). Despite significant advances in technology and hygienic practices at all levels of chicken production, salmonellosis poses an unrelenting threat to human and animal health. It is caused by a large group of bacteria of the genus *Salmonella* under the family *Enterobacteriaceae* (OIE, 2018). There are more than 2,600 serotypes of Salmonella broadly categorized into host‐restricted, host‐adapted and generalist based on their host specificity, virulence, phage typing, etc (Mezal et al., 2014). Among them, *Salmonella Gallinarum* and *Salmonella Pullorum* are host‐restricted non‐motile serovars of chicken. However, chickens commonly harbour other generalist non‐typhoidal (NT) serovars of public health significance such as *S*. *Typhimurium*, *S*. *Enteritidis*, *S*. *Heidelberg* and *S*. *Newport* (Wray et al., 1996). These non‐hosts adapted serovars rarely cause clinical diseases in chickens, but they can be transmitted to humans through consumption of contaminated eggs and/or meat (Wray et al., 1996). The *Salmonella* serovar *Gallinarum* may be divided into biovars *Gallinarum* and *Pullorum*, which are, respectively, responsible for the fowl typhoid and the pullorum disease of chickens, and are widely distributed throughout the world, especially in developing countries (Barbour et al., 2015). Pullorum disease occurs in chicks during their first few days of life, and fowl typhoid is a disease of mature fowls that drops egg production (OIE, 2018).

Antibiotics have been used in livestock and poultry to treat infections and improve feed efficiency (Hutchinson et al., 1991) as well as to control and prevent infections (Tollefson & Miller, 2000). Poultry products are one of the most commonly consumed products worldwide, but lots of essential antibiotics are used in many countries during its production, threatening the safety of these products (through antimicrobial residues) and the increased possibility of development and spread of microbial resistance in poultry settings (Agyare et al., 2018). Antimicrobial resistance (AMR) is a burgeoning problem for public health, particularly with the introducing of multi‐drug‐resistant (MDR) microorganisms. In developing countries like Bangladesh, antimicrobials are used not only for therapeutic purposes but also for growth promotion in the poultry industry. Although *S*. *Gallinarum* and *S*. *Pullorum* cause diseases only in chicken, the emergence of antimicrobial resistance among these serovars can be horizontally transmitted into other non‐typhoidal zoonotic serovars. Antimicrobial‐resistant zoonotic bacteria are of particular concern as they may impede effective treatment regimes in humans (Prestinaci et al., 2015). Therefore, determining the nature and extent of AMR found in poultry in *Salmonella* is essential. Antibacterial sensitivity tests usually are performed to select the suitable antibacterial agents for the effective therapeutic purpose of salmonellosis; however, due to the recent emergence of MDR *Salmonella* strain, antibiotic treatment for salmonellosis is getting difficult (Kuehn, 2019; Nair et al., 2018).

Salmonellosis is important as both a cause of clinical disease in commercial poultry that hindered the development of the poultry industry in Bangladesh and as a source of human food‐borne zoonotic diseases (Mahmud et al., 2011; Waltman et al., 2008). For proper control and management of salmonellosis, it is necessary to determine its status at the farm level. Isolation, identification and characterization of the particular aetiological agent are essential for a better understanding of a disease situation in a particular area (Ahmed et al., 2008). Prevention and control of salmonellosis require to identify it's antimicrobial resistance pattern. Therefore, the present study was undertaken with the objectives (1) to determine the prevalence of *Salmonella*; it's isolation and identification from apparently healthy chickens, (2) to determine the antimicrobial resistance pattern of the isolates.

## 2 MATERIALS AND METHODS

**FIGURE 1:**
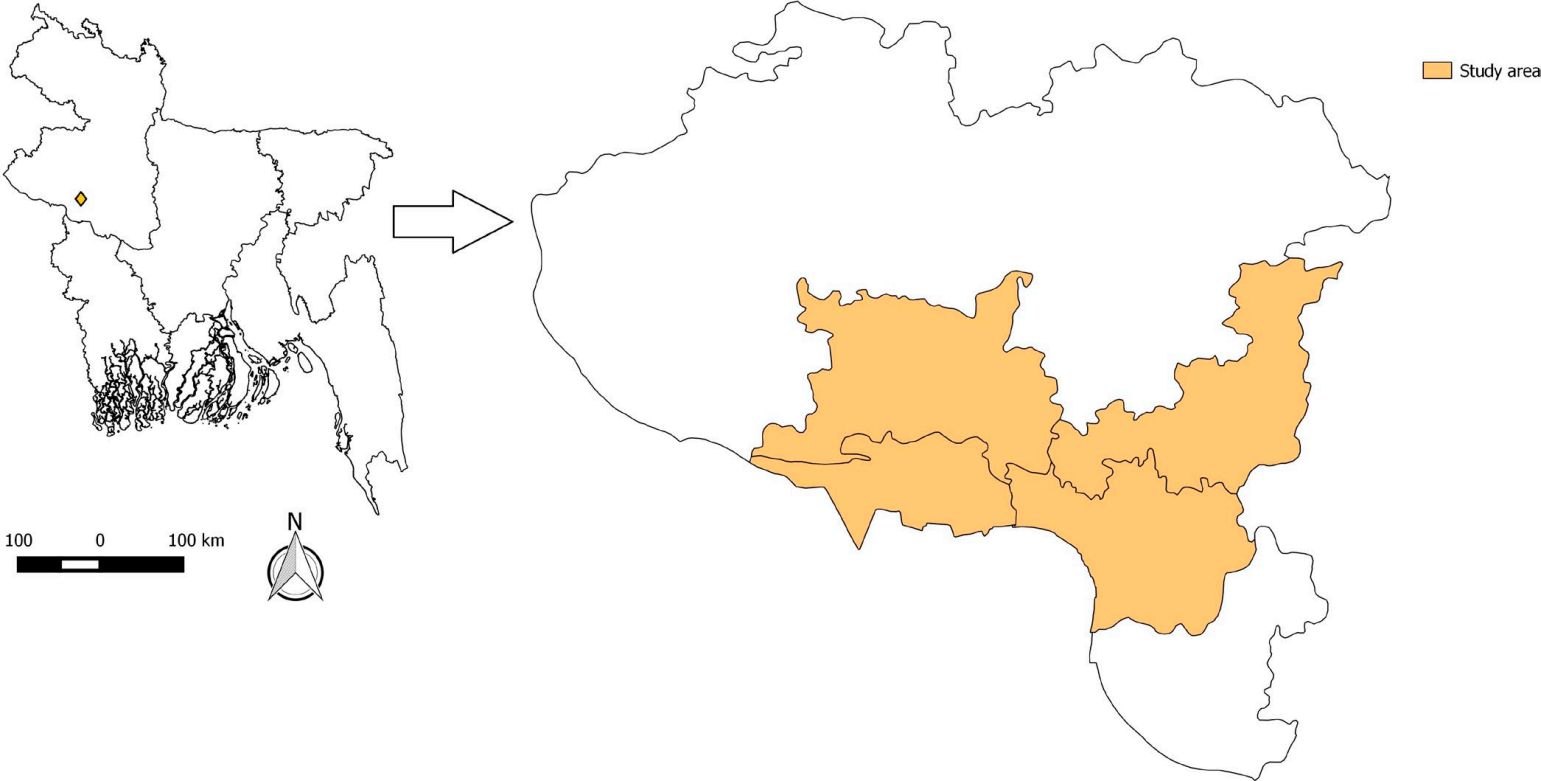
Map of Bangladesh showing the location of Rajshahi. Inset showing the Rajshahi district with different study location, 2016

**FIGURE 2:**
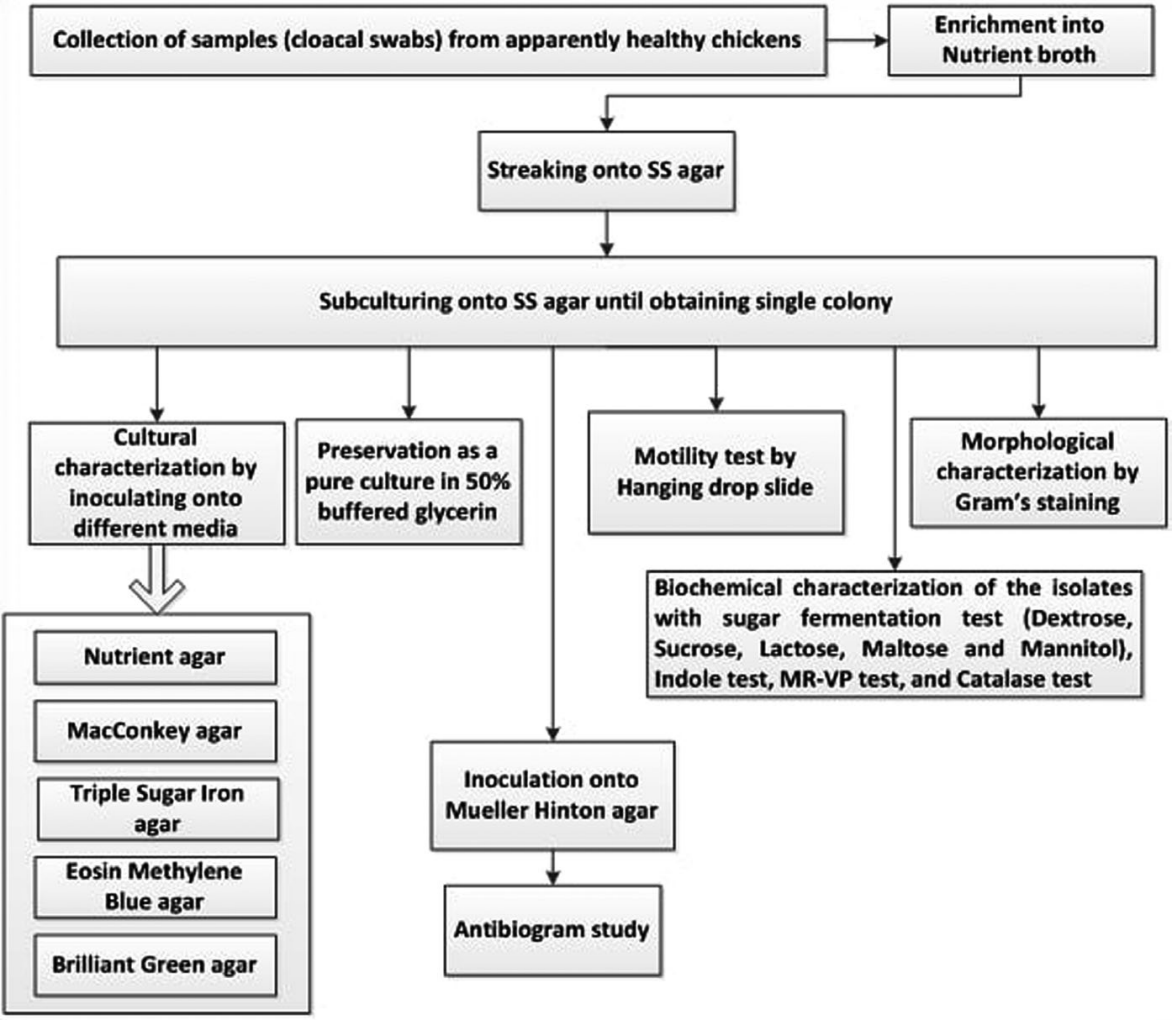
Flow chart of the experimental design for non‐typhoidal *Salmonella* in chicken in Rajshahi, Bangladesh, 2016

We conducted the study from January to June 2016 in 24 randomly selected poultry farms of three different study areas, namely Puthia *Upazila* (sub‐districts), Charghat *Upazila* and Rajshahi City Corporation of the Rajshahi district of Bangladesh (Figure 1). We drew an experimental design for conducting the study following the different steps (Figure 2).

### 2.1 Sampling

We collected a total of 120 cloacal swab samples from the apparently healthy chickens of the selected farms. An equal number of samples (40) were collected from each of the three study areas, and among these, 60 samples from broiler farms and 60 samples from layer farms. More specifically, five samples were collected from each of the 24 farms.

Samples were collected from the mucosa of the cloacal opening of both broiler and layer chickens. A sterile swab stick moistened with sterile normal saline water was inserted into the chicken's cloaca, collected the sample and then placed in sterile vials having Stuart's transport medium in the icebox. The swabs were collected randomly and aseptically then transferred immediately to the laboratory.

We recorded the following data during sample collection: flock size, rearing system, feed type, vaccination, biosecurity, age of birds and type of birds (broiler/layer). We recognized those flocks as larger flock, which had more than 1,000 chicken and smaller flock with less than 1,000 chicken. We termed those feed as a 'loose feed' that were formulated readily in the farm by mixing different feed ingredients and a 'ready feed' that brought from commercially available feed company in the form of mash, crumble or pellets and fed directly to chickens without mixing any ingredients in the farm.

### 2.2 Cultivation and isolation of non‐typhoidal *Salmonella* from the cloacal swab

#### 2.2.1 Cultivation of *Salmonella*

Each swab was inoculated separately into the freshly prepared nutrient broth and marked appropriately. Then, these were incubated at 37°C for 24 hr aerobically in a bacteriological incubator. The incubated tubes were then examined for bacteria growth. After that, the organism was inoculated into Salmonella‐Shigella (SS) agar plate and incubated at 37°C overnight. The colonies on primary culture were subcultured by the streak plate method until the pure culture with homogenous colonies were obtained (Cheesbrough, 1987). Media such as Nutrient agar (NA), Salmonella‐Shigella (SS) agar, MacConkey agar, Eosin methylene blue agar (EMBA), Triple sugar iron (TSI), Simmons’ citrate agar (SCA) and Brilliant green agar (BGA) were used for subcultures.

#### 2.2.2 Isolation of *Salmonella*

Salmonella inoculum was inoculated in SS agar by streak plate technique to obtain isolated colonies (Cheesbrough, 1987). The method was repeated as many times as necessary to obtain a culture containing singe colonies only and usually at least two or more times to ensure purity.

#### 2.2.3 Identification of *Salmonella*

We identified *Salmonella* based on their cultural characteristics, colony character, morphology, Gram's staining, motility and biochemical test. Shape, size, surface texture, edge, elevation, colour and opacity were observed and recorded after 24 hr of incubation for characterizing colony morphology. The Salmonella colonies were stained using Gram's staining method (Merchant & Packer, 1967). The motility test was done for the separation of motile and non‐motile *Salmonella* (Cheesbrough, 1987).

#### 2.2.4 Characterization of *Salmonella*

We characterized the isolated *Salmonella* by using the following biochemical test: catalase test, sugar fermentation test (Dextrose, Sucrose, Lactose, Maltose and Mannitol), TSI test, Simon citrate agar test, Indole test and MR‐VP test (Cown, 1985).

#### 2.2.5 Antibiogram study of the isolated *Salmonella*

We performed an antibiotic susceptibility test of *Salmonella* isolates against eight antimicrobial agents by disc diffusion methods, as stated by the guidelines of Clinical and Laboratory Standard Institute (CLSI, 2012). A total of 20 samples were used for the antibiogram study. Sensitivity and resistance of the isolates were determined against streptomycin, penicillin, gentamicin, ampicillin, ciprofloxacin, amoxicillin, nalidixic acid and sulfamethoxazole‐trimethoprim. The antimicrobial discs were dispensed onto the surface of Muller Hinton agar plates using sterile forceps, keeping a distance of about 1cm apart. Within 30 min after applying the discs, the plates were incubated at 37°C for 18 hr in an inverted position. Three or four different discs were placed on one plate. Each disc was pressed down to ensure complete contact with the agar surface. After incubation, each plate was examined. The diameters of the zone of inhibition were measured using a meter ruler. The zone margin was taken as the area showing no obvious, visible growth that can be detected with the unaided eye. The zone of inhibition was interpreted as sensitive, intermediate and resistant, according to CLSI guideline (CLSI, 2012). Any isolate resistant to at least three classes of antimicrobials were considered as multidrug resistant (Magiorakos et al., 2012). The zone of diameter interpreted as the standard for *Salmonella* is mentioned in Table 1.

**TABLE 1:** Inhibition zone diameter for non‐typhoidal *Salmonella* in chicken in different farms of Rajshahi, Bangladesh, 2016

Antibiotic disc	Resistance	Intermediate	Sensitive
Streptomycin	≤11	12–14	≥15
Penicillin	≤11	12–21	≥22
Gentamicin	≤12	13–14	≥15
Ampicillin	≤13	14–16	≥17
Ciprofloxacin	≤15	16–20	≥21
Amoxicillin	≤13	14–16	≥18
Nalidixic acid	≤13	14–18	≥19
Sulfamethoxazole‐trimethoprim	≤10	11–15	≥16

Note

≤Less than or equal, ≥Greater than or equal.

#### 2.2.6 Maintenance of stock culture

For further study, it was necessary to preserve the *Salmonella* isolates. For this purpose, pure culture of isolated *Salmonella* was preserved in 50% sterile buffered glycerine and stored at −20°C.

### 2.3 Data analysis

We calculated the prevalence of non‐typhoidal *Salmonella* at the farm level by dividing culture‐positive samples by the total number of tested samples. We also performed bivariate logistic regression analysis to identify the association between the non‐typhoidal *Salmonella* and the variables of interest. The odds ratio (OR) with 95% confidence interval (CI) at 0.05 significance level was estimated to measure the degree of association. Data collected from a questionnaire survey (from the respective study farm) and laboratory study were entered into a Microsoft Excel sheet and analysed using STATA version 13 (Stata Corp & L., 2013).

## 3 RESULTS

### 3.1 Prevalence of non‐typhoidal *Salmonella*

Of the total 120 samples tested, 49 (41%) [95% confidence interval (CI): 31.95%–50.17%] were positive for *Salmonella* (Table 2). The prevalence of *Salmonella* was 42% in broiler chicken (*n* = 25) [95% confidence interval (CI): 29.06%–55.12%] and 40% in layer chicken (*n* = 24) [95% confidence interval (CI): 27.56%–53.46%] (Table 3). Sample collected from Rajshahi city (OR = 1.37, 95% CI: 0.50–3.73) and Puthia *Upazila* (OR = 1.51, 95% CI: 0.56–4.12) was more likely to be positive for *Salmonella* as compared to Charghat *Upazila*. Sample collected from the farms using loose feed (OR = 1.26, 95% CI: 0.49–3.29) for chicken was more likely to be positive for *Salmonella* than those used ready feed (Table 4). The prevalence of *Salmonella* was higher in the larger flock (47.7%) compared to the smaller flock (32.7%).

**TABLE 2:** Prevalence of non‐typhoidal *Salmonella* in chicken in different farms of Rajshahi, Bangladesh, 2016

Farm No	Region of farms	Flock size	Rearing system	No. of samples tested (*n* = 120)	No. of positive case (*n* = 49)	Overall percentage
01	RC_1_	700	Liter	5	2	41
02	RC_2_	3,000	Liter	5	3	
03	RC_3_	8,000	Liter			
04	RC_4_	1,200	Liter	5	2	
05	RC_5_	700	Liter	5	1	
06	RC_6_	2,000	Liter	5	3	
07	RC_7_	3,000	Liter	5	2	
08	C_1_	500	Liter	5	3	
09	C_2_	600	Liter	5	3	
10	C_3_	1,800	Liter	5	4	
11	C_4_	1,065	Liter	5	1	
12	C_5_	2,500	Liter	5	0	
13	C_6_	300	Liter	5	0	
14	C_7_	2,000	Liter	5	2	
15	C_8_	1,000	Liter	5	1	
16	P_1_	1,500	Liter	5	4	
17	P_2_	12,000	Liter	5	1	
18	P_3_	300	Liter	5	3	
19	P_4_	500	Liter	5	2	
20	P_5_	700	Liter	5	1	
21	P_6_	1,500	Liter	5	4	
22	P_7_	500	Liter	5	0	
23	P_8_	1,200	Liter	5	3	
24	RC_8_	800	Liter	5	2	

Abbreviations: C, Charghat; RC, Rajshahi City, P, Puthia.

**TABLE 3:** Prevalence of non‐typhoidal *Salmonella* in broiler and layer chicken in Rajshahi, Bangladesh, 2016

Types of chicken	No of samples tested (*n* = 120)	No. of positive case (*n* = 49)	Prevalence (%)	Overall prevalence (%)
Broiler	60	25	41.7	41
Layer	60	24	40.0	

**TABLE 4:** Factors for the prevalence of non‐typhoidal *Salmonella* in chicken in Rajshahi, Bangladesh, 2016

	No of samples tested (*n* = 120)	Sample +ve	Sample‐ve	OR (95% CI)	*P* value
Sample collection area					
Charghat	40	14	26	Ref	
Rajshahi city	40	17	23	1.37 (0.50–3.73)	0.491
Puthia	40	18	22	1.51 (0.56–4.12)	0.361
Types of feeds used					
Ready feed	90	38	52	Ref	
Loose feed	30	11	19	1.26 (0.49–3.29)	0.591

### 3.2 Cultural findings

**FIGURE 3:**
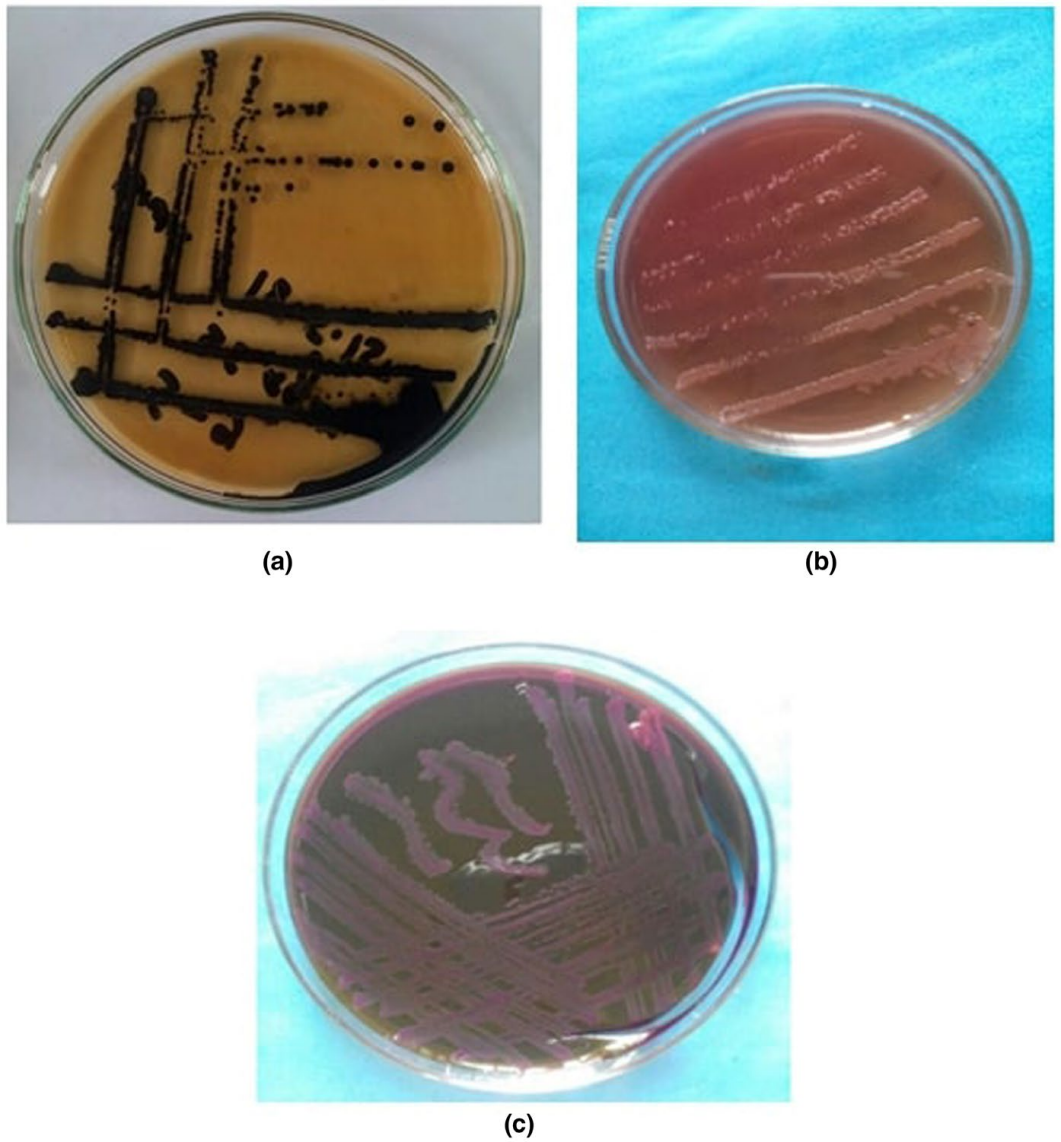
Cultural characteristics on different solid media for non‐typhoidal *Salmonella* isolates from chicken in Rajshahi, Bangladesh, 2016

After cultural examination, we found that the positive samples showed the characteristic colonies in different media such as SS, BGA, MAC, TSI, NA and EMBA (Figure 3). *Salmonella* isolated from the cloacal swabs produced a black centred, smooth and small round colony on SS agar, whereas a translucent pink colony surrounded by a pink zone on BG agar. On MacConkey agar, colourless, smooth, transparent and raised colony was produced. On TSI agar, black colour colony against a yellowish background was raised. Translucent, opaque and smooth colony on NA and colourless, transparent or amber colour was raised on EMB agar.

### 3.3 Staining and motility test

**FIGURE 4:**
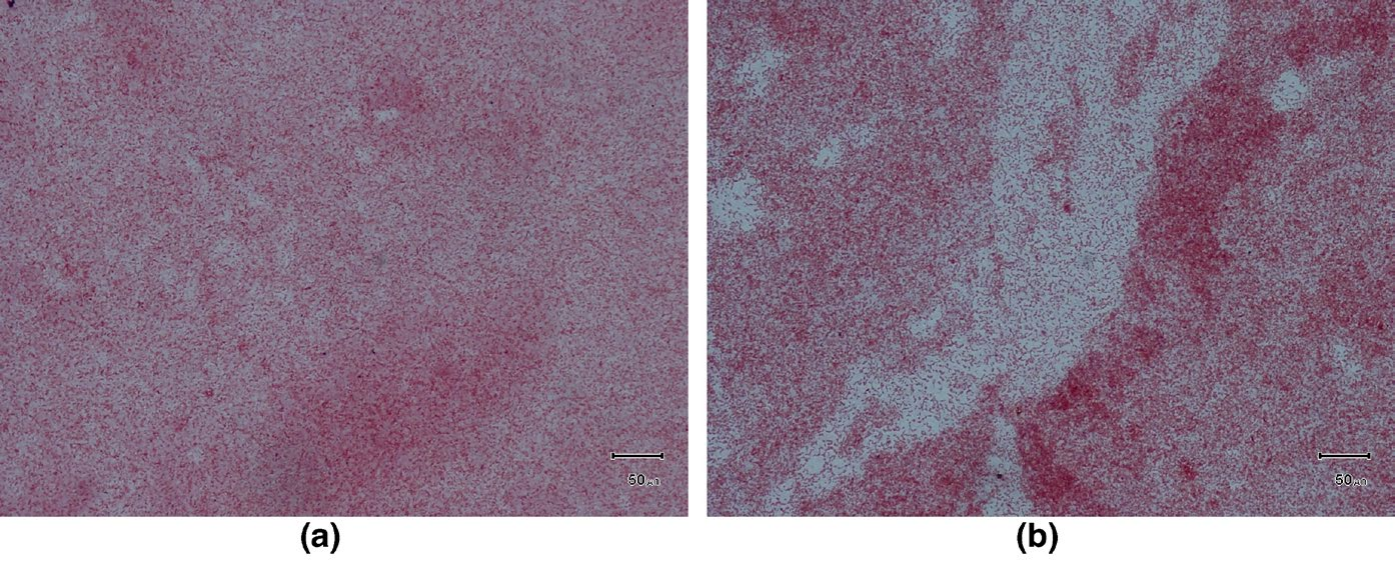
Gram staining tests for isolated non‐typhoidal *Salmonella sp*. from (a) Broiler and (b) Layer chicken in Rajshahi, Bangladesh, 2016 [light microscope (100×)]

Morphological characterization revealed that the isolates were Gram‐negative, short, plump, rod‐shaped organism, arranged in a single or paired. In the motility test, we found that they were non‐motile (Figure 4).

### 3.4 Biochemical tests

**FIGURE 5:**
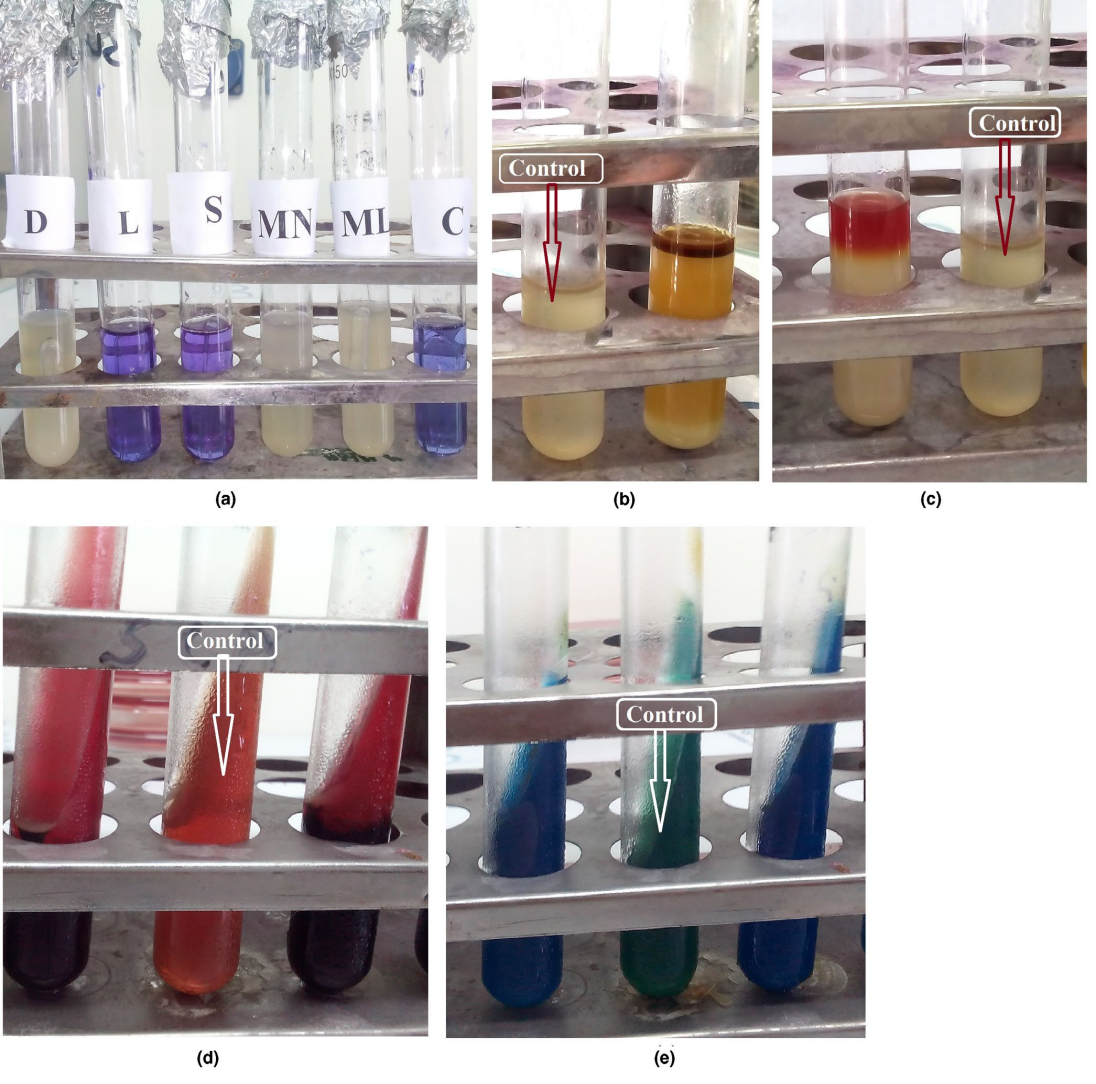
Biochemical test for non‐typhoidal *Salmonella* from chicken in Rajshahi, Bangladesh, 2016. (a) Fermentation reaction for *Salmonella* with five basic sugars (D, Dextrose; ML, Maltose; L, lactose; S, Sucrose; MN, Manitol; C, Control). (b) Indole test for *Salmonella* (negative) did not produce red colour ring. (c) *Salmonella* showed positive result in MR test. (d) *Salmonella* produced H_2_S, evidenced by black colour for H_2_S production. (e) *Salmonella* showed positive result in MR test

After biochemical examination, we observed that all of the isolates fermented dextrose, maltose and mannitol and produced acid and gas but did not ferment sucrose and lactose. Additionally, all the isolates were positive to the methyl red test, catalase, TSI agar slant reaction and Simmon's citrate agar slant reaction, but negative to indole test and Voges‐Proskauer test (Table 5 and Figure 5).

**TABLE 5:** Results of biochemical test for non‐typhoidal *Salmonella* isolated from chicken in different farms of Rajshahi, Bangladesh, 2016

No of samples tested	Fermentation with five basic sugars		Other biochemical tests		Interpretation
120	Name of sugar	Results	Name of test	Results	*Salmonella*
	Dextrose	+ (AG)	Catalase	+	
	Maltose	+ (AG)	Indole	−	
	Lactose	−	MR	+	
	Sucrose	−	VP	−	
	Mannitol	+ (AG)	Simmons’ citrate agar slant reaction	Changed colour from green to intense blue	
			TSI agar slant reaction	Changed colour from yellow to black	

Abbreviations: −, negative reaction; +, positive reaction; AG, production of acid and gas; MR, Methyl red;TSI, Triple sugar iron; VP, Voges‐Proskaure.

### 3.5 Antibiotic sensitivity test

**FIGURE 6:**
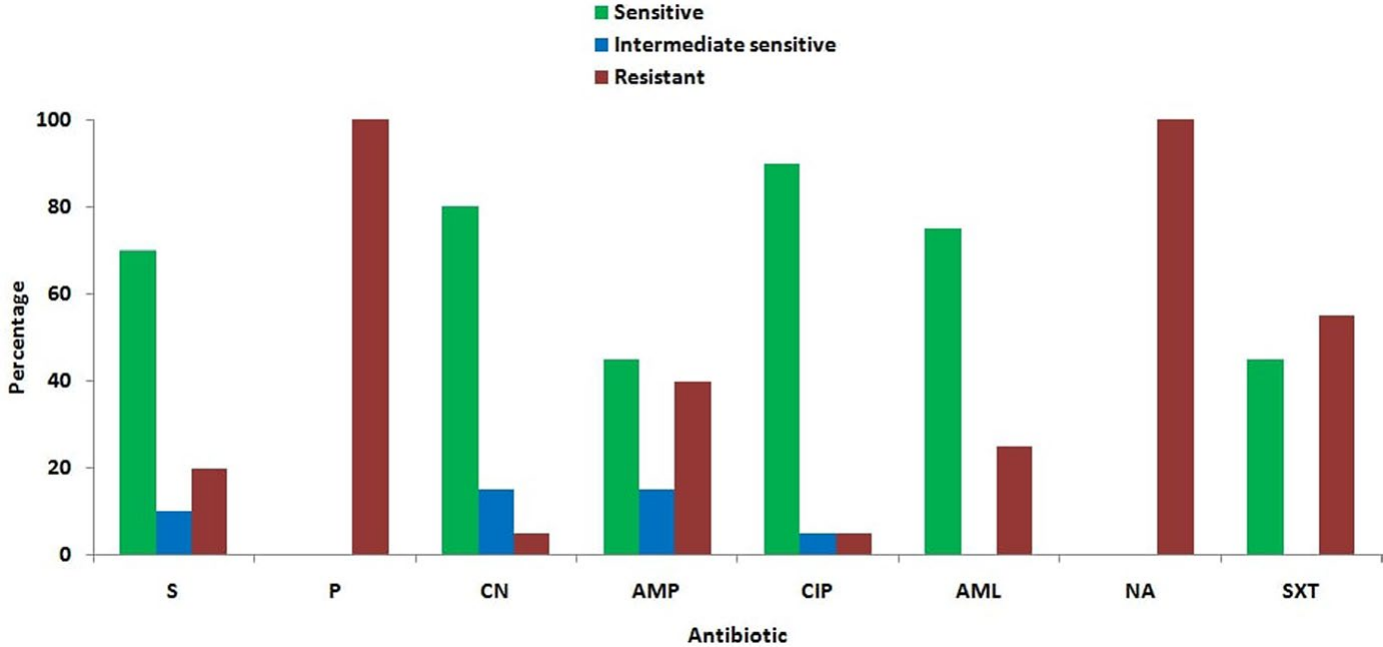
Antibiotic sensitivity and resistant patterns of *Salmonella* from chicken in Rajshahi, Bangladesh, 2016. AML, Amoxicillin; AMP, Ampicillin; CIP, Ciprofloxacin; CN, Gentamicin; NA, Nalidixic acid; P, Penicillin; S, Streptomycin; SXT, Sulfamethoxazole‐trimethoprim.

**FIGURE 7:**
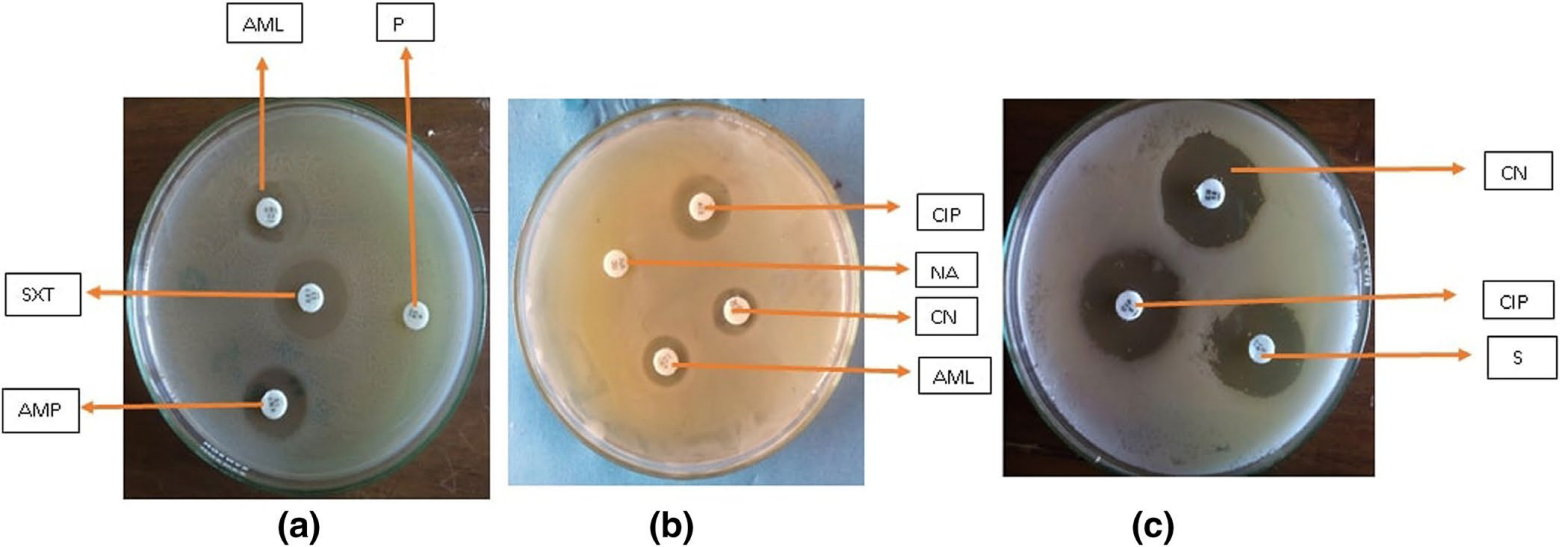
Antibiotic sensitivity of non‐typhoidal *Salmonella* isolates from chicken in Rajshahi, Bangladesh, 2016

From the antibiogram study against eight different antibiotics, it was revealed that the resistance patterns for *Salmonella* isolates were 100% to penicillin and nalidixic acid, 55% to sulfamethoxazole‐trimethoprim, 40% to ampicillin, 25% to amoxicillin, 20% to streptomycin and 5% to gentamicin and ciprofloxacin. On the other hand, the sensitivity pattern of the isolates was 90% to ciprofloxacin, 80% to gentamicin, 75% to amoxicillin, 70% to streptomycin and 45% to sulfamethoxazole‐trimethoprim and ampicillin (Figures 6 and 7).

## 4 DISCUSSION

In this study, we determined the prevalence of poultry‐specific non‐typhoidal *Salmonella and* it's antibiotic susceptibility patterns from apparently healthy chickens collected from different poultry farms of Bangladesh. The overall prevalence of non‐typhoidal *Salmonella* in this study was 41%. This finding was almost in agreement with the report of (Alebachew & Mekonnen, 2013), who reported 41.9% *Salmonella* infection among chicken flock in Jimma town, Ethiopia (Alebachew & Mekonnen, 2013). However, the present finding was lower than the findings of Parbati et al. (2017) and Naurin et al. (2012), who reported 53.33% and 52% prevalence of *Salmonella* in chickens, respectively (Naurin et al., 2012; 2017). Our findings were higher than the findings of Bhuyan et al. (2010), who recorded a 16.52% prevalence of *Salmonella* in poultry. Similarly, Alam et al. (2003) reported a 23.8% prevalence of *Salmonella* infection in poultry in the Dinajpur district of Bangladesh. The prevalence may vary due to differences in the origin of samples, the technique used or due to different environmental conditions. There was a difference in the prevalence of *Salmonella* infection in different areas in our study. We found a higher prevalence of non‐typhoidal *Salmonella* in chicken, providing loose feed than those provided with ready feed. Research has shown that changes in feed by modifying ingredients and composition of nutrients have an effect on the sensitivity of chickens to *Salmonella* infection (Vandeplas et al., 2010). The highest prevalence was at Puthia Upazila (15%), followed by the Rajshahi city corporation area (14.2%) and Charghat Upazila (11.7%). Similarly, Bhuyan et al. (2010) reported a variation in the prevalence of *Salmonella* in different areas, such as in Gazipur (20%), Manikgonj (16%) and Saver (15%) of Bangladesh. The prevalence of *Salmonella* was 41.7% in broiler and 40% in the layer. This finding was supported by other studies where the prevalence of *Salmonella* in broiler and layer was 41.3% and 46.2%, respectively (Alebachew & Mekonnen, 2013). However, a higher prevalence of *Salmonella* was found in a study where the prevalence was 71.11% in broiler and 38.8% in layer chickens (Naurin et al., 2012). Flock size also influenced the prevalence of *Salmonella* infection in our study. We found a higher prevalence in the larger flock (47.7%) compared to the smaller flock (32.7%). This finding is in agreement with the findings of another study in Bangladesh where they reported a higher prevalence (34.2%) of *Salmonella* in large flocks (≥5,001 birds) and lower prevalence (21.3%) in small flocks (≤1,000 birds) (Hossain et al., 2010). The highest infection rate in larger flocks may be due to the high flock density, which facilitates the easy spread of any infection.

Emerging antimicrobial resistance in the food‐borne bacterial isolates is a major public health concern. Over the past 30 years, extensive use of antibiotics in livestock has led to increased antibiotic resistance in various bacterial strains (Mölstad et al., 2017). *Salmonella* is one of the MDR bacteria, showing resistance to ampicillin, streptomycin, chloramphenicol, sulfonamides and tetracycline (Guilfoile & Alcamo, 2007). The antibiotic sensitivity patterns in our study showed that the non‐typhoidal *Salmonella* isolates were 100% resistant to penicillin and nalidixic acid, followed by sulfamethoxazole‐trimethoprim (55%), ampicillin (40%) and amoxicillin (25%). Similar findings have been documented in other studies where resistance to penicillin and nalidixic acid was 100% (Bhuyan et al., 2010; Seyyedeh et al., 2013). A study in Bangladesh showed that *Salmonella* strains were 100% resistant to nalidixic acid (2017). We found that *Salmonella* isolates were sensitive to ciprofloxacin (90%), gentamicin (80%), amoxicillin (75%), streptomycin (70%), followed by ampicillin (45%) and sulfamethoxazole‐trimethoprim (45%). Ciprofloxacin sensitivity (100%) to *Salmonella* isolates had also been documented by other studies( Nesa et al., 2011; Obi & Ike, 2015; Ramya & MadhavaraoTirupati, 2013; Seyyedeh et al., 2013). We also found that the isolates were 80% sensitive to gentamicin, which was similar to other studies where they found 90%, 92.8% and 100% sensitivity, respectively (Bhuyan et al., 2010; Obi & Ike, 2015; Ramya & MadhavaraoTirupati, 2013). We found that the sensitivity pattern for streptomycin was 70%. Similar findings have also been reported in other studies where the isolates were 80% sensitive to streptomycin( Ramya & MadhavaraoTirupati, 2013).

The test organisms in our study were Gram‐negative short, rod‐shaped and mostly occurred singly or occasionally paired, which also corresponded to morphological characters of *Salmonella* as described in other study( Cheesbrough, 1987). In most instances, we found that the test organisms were non‐motile. *Salmonella Gallinarum* and *Salmonella Pullorum* are non‐motile, whereas other poultry *Salmonella* spp. are found to be motile (Cheesbrough, 1987; Christensen et al., 1993). We found the organism was grown on a different media where they produced circular, smooth, opaque and translucent colonies on NA; black centred and small round on SS agar; translucent pink colony surrounded by a pink zone on BGA; pale, smooth, transparent and raised colonies on MacConkey agar; large, colourless colonies on EMB agar media and on TSI agar slant, black colony against a yellowish background were produced which was corresponded to the findings of others studies (Buxton & Fraser, 1977; Cheesbrough, 1987). The isolates fermented dextrose, maltose, and mannitol and produced both acid and gas, which was corresponded to the findings of others (Hasan et al., 2010; Merchant & Packer, 1967). Both indole and Voges‐Proskauer tests were negative, but methyl red, catalase, TSI agar slant reaction and Simmon's citrate agar slant reaction were positive, which are almost similar to the findings of Buxton & Fraser (1977).

## 5 CONCLUSION

This study's results evidenced the occurrence of host‐specific *Salmonella* serovars in commercial chicken production in Bangladesh, indicating that even apparently healthy chickens could be an important source of salmonellosis for chickens. Proper hygiene and disinfection practices at the farm‐level could be effective in the overall reduction of *Salmonella*. A considerable proportion of *Salmonella* isolates was found to be resistant to different classes of antimicrobial drugs that could have a significant impact on public health if the resistance mechanisms are transferred into other serovars of zoonotic significance. Therefore, the regulation of the irrational use of antimicrobials in chickens must be addressed, including the restriction of antimicrobial supply in the illegal market.

## CONFLICTS OF INTEREST

The authors declare that they have no conflicts of interest.

## AUTHOR CONTRIBUTION

**Bindu Rani Sarker:** Conceptualization; Data curation; Formal analysis; Funding acquisition; Investigation; Methodology; Project administration; Resources; Software; Validation; Writing‐original draft; Writing‐review & editing. **Sumon Ghosh:** Conceptualization; Formal analysis; Investigation; Methodology; Project administration; Resources; Software; Supervision; Validation; Visualization; Writing‐review & editing. **Sukanta Chowdhury:** Formal analysis; Methodology; Software; Writing‐review & editing. **Avijit Dutta:** Methodology; Validation; Visualization; Writing‐original draft; Writing‐review & editing. **Liton Chandra Deb:** Formal analysis; Methodology; Software; Writing‐review & editing. **Bidhan Krishna Sarker:** Formal analysis; Investigation; Methodology; Supervision; Writing‐review & editing. **Tania Sultana:** Data curation; Investigation; Methodology; Supervision; Visualization; Writing‐review & editing. **K. M Mozaffor Hossain:** Conceptualization; Formal analysis; Investigation; Methodology; Project administration; Software; Supervision; Writing‐review & editing.

### Peer Review

The peer review history for this article is available at https://publons.com/publon/10.1002/vms3.440.
